# Impact of obesity on outcomes of patients with acute respiratory distress syndrome: a retrospective analysis of a large clinical database

**DOI:** 10.1007/s00063-023-01042-7

**Published:** 2023-08-16

**Authors:** Qiyan Lou

**Affiliations:** Department of Respiratory Medicine, Zhuji Affiliated Hospital of Wenzhou Medical University, No. 9 Jianmin Road Taozhu Street, 311800 Zhuji, China

**Keywords:** Body mass index, ARDS, Mortality, Cox regression, Body Mass Index, Atemnotsyndrom, Mortalität, Cox-Regression

## Abstract

**Objective:**

To evaluate the link between obesity and mortality in patients with acute respiratory distress syndrome (ARDS).

**Methods:**

We performed a retrospective cohort study of a large clinical database. A Cox proportional hazards regression model was used to calculate the hazard ratio (HR) and 95% confidence interval (CI) for the relationship between body mass index (BMI) and mortality. The primary endpoint was 30-day death rate and the secondary endpoints were 90-day and 1‑year mortality.

**Results:**

Overall, 418 patients with ARDS were enrolled in the study, including 185 women and 233 men (age: 70.7 ± 44.1 years; BMI: 28.7 ± 8.1 kg/m^2^). Compared with patients with normal weight, obese patients were younger (60.1 ± 13.7, *p* = 0.003) and a higher percentage of these patients were women (51.3% vs. 49.0%, *p* = 0.001). The HRs (95% CI) of 30-day mortality in the underweight, overweight, and obese populations were 1.82 (0.85, 3.90), 0.59 (0.29, 1.20), and 3.85 (1.73, 8.57), respectively, after adjustment for other confounding factors. A similar pattern was also seen for death after 90 days and after 1 year. A U-shaped association between BMI and 30-day mortality was discovered by curve fitting.

**Conclusion:**

Obesity had a significant impact on the short- and long-term mortality in patients with ARDS. There was a U-shaped relationship between BMI and mortality, while a higher BMI was associated with an increased risk of death in patients with ARDS.

## Abstract (de)

**Ziel:**

Ziel der Arbeit war es, den Zusammenhang zwischen Adipositas und Mortalität bei Patienten mit akutem respiratorischem Disstress-Syndrom (ARDS) zu untersuchen.

**Methoden:**

Zu diesem Zweck wurde eine retrospektive Kohortenstudie einer großen klinischen Datenbank durchgeführt. Mithilfe eines Proportional-Hazards-Regressionsmodells nach Cox wurden die Hazard Ratio (HR) und das 95 %-Konfidenzintervall (95 %-KI) für die Beziehung zwischen Body Mass Index (BMI) und Mortalität berechnet. Primärer Endpunkt war die 30-Tage-Mortalität, während die sekundären Endpunkte aus der 90-Tage-Mortalität sowie der Mortalität nach einem Jahr bestanden.

**Ergebnisse:**

Insgesamt wurden 418 Patienten mit ARDS in die Studie einbezogen, davon 185 w. und 233 m. (Alter: 70,7 ± 44,1 Jahre; BMI: 28,7 ± 8,1 kg/m^2^). Im Vergleich zu normalgewichtigen Patienten wiesen die adipösen Patienten ein geringeres Alter auf (60,1 ± 13,7; *p* = 0,003), außerdem war der Frauenanteil in dieser Patientengruppe höher (51,3 vs. 49,0%; *p* = 0,001). Die HR (95 %-KI) der 30-Tage-Mortalität betrug in der untergewichtigen Population 1,82 (0,85; 3,90); in der übergewichtigen 0,59 (0,29; 1,20) bzw. in der adipösen Population 3,85 (1,73; 8,57) nach Berücksichtigung anderer Störfaktoren. Ein ähnliches Muster fand sich auch für den Tod nach 90 Tagen und nach einem Jahr. Durch Kurvenanpassung wurde ein U‑förmiger Zusammenhang zwischen BMI und 30-Tage-Mortalität festgestellt.

**Schlussfolgerung:**

Adipositas hat einen erheblichen Einfluss auf die kurz- und langfristige Mortalität bei Patienten mit ARDS. Es fand sich ein U‑förmiger Zusammenhang zwischen BMI und Mortalität, dabei ging ein höherer BMI mit einem erhöhten Sterberisiko bei Patienten mit ARDS einher

## Introduction

Acute respiratory distress syndrome (ARDS) is an acute inflammatory lung injury that is accompanied by severe hypoxemia, increased lung weight, increased pulmonary vascular permeability, and loss of aerated lung tissue [1, 2]. In the intensive care unit (ICU), ARDS is a leading cause of death and a major healthcare burden; for example, the Global Impact of Severe Acute Respiratory Failure (LUNG SAFE) research found that over 34% of patients died from the condition [3]. Despite improvements in our knowledge of the processes underlying ARDS, the mortality rate improvement has been far from satisfactory [4]. In order to help doctors make treatment decisions and identify individuals at high risk, it is necessary to find prognostic indicators of ARDS [5].

Obesity is considered an epidemic and is brought on by people’s increasing desire for calorie-dense foods and sedentary lifestyles. The increased morbidity of conditions such as cancer, hypertension, diabetes, coronary heart disease, and others is a result of obesity and overweight [6, 7]. Recent research, however, has uncovered a “obesity paradox” in which obesity may potentially act as a preventative measure for certain illnesses, such as chronic renal disease and heart failure [8–10]. Numerous studies [11–14] have looked at the connection between obesity and ARDS outcomes. According to a meta-analysis by Yue et al. [15] that pooled data from five studies and 6268 individuals, being underweight was linked to greater mortality when compared to being at a normal weight, while reduced mortality was more likely to be caused by obesity and morbid obesity. These studies did not take into account a number of significant confounding variables, including alcohol use status, illness severity, and cardiovascular disease. However, the existence of the obesity paradox in relation to ARDS has remained debatable.

Therefore, using real-world data from the most recent Multiparameter Intelligent Monitoring in Intensive Care (MIMIC-III) database, we sought to evaluate the relationship between obesity and the chances of short- and long-term death in patients with ARDS.

## Methods

### Study design and population

The study is reported according to the Strengthening the Reporting of Observational Studies in Epidemiology (STROBE) statement [16].

The MIMIC-III version 1.4 was used in our retrospective investigation [17, 18]. The database contains information on 58,976 patients who were hospitalized in the intensive care unit (ICU) of Beth Israel Deaconess Medical Center between 2001 and 2016. The Massachusetts Institute of Technology and Beth Israel Deaconess Medical Center Institutional Review Boards gave their approval of the project.

In the MIMIC-III version 1.4, we initially screened 58,976 patients. Participants were included if any of the following criteria were met: (1) a confirmed diagnosis of ARDS (hypoxemic respiratory failure requiring intubation and PaO_2_/FiO_2_ ≤ 200 mm Hg; bilateral infiltrates on chest radiographs; and absence of left atrial hypertension [19]); (2) body mass index (BMI) was determined on the first day of admission; (3) hospital stays exceeded 2 days; and (4) age ≥ 18 years old. Missing data of more than 5% was an exclusion criterion.

### Data collection

Age, gender, and ethnicity data were gathered, along with clinical traits, comorbidities, and rating systems. Vital indicators comprised heart rate, SPO_2_, systolic blood pressure (SBP), and diastolic blood pressure (DBP) for 24 h after ICU admission. Comorbid conditions included high blood pressure, type 2 diabetes, coronary heart disease, chronic heart failure, atrial fibrillation, and acute kidney injury (AKI). The Glasgow Coma Scale (GCS) score, the Sequential Organ Failure Assessment (SOFA; [20]), the Simplified Acute Physiology Score II (SAPS II), and the Acute Physiology and Chronic Health Evaluation (APACHE) III [21] were all used [22].

The BMI was computed using the patient’s height and weight as of the first day following admission. According to the World Health Organization BMI classifications [23], BMI was classified into four categories: underweight (BMI 18.5 kg/m^2^), normal weight (BMI: 18.5–25 kg/m^2^), overweight (BMI: 25–30 kg/m^2^), and obese (BMI > 30 kg/m^2^).

### Definition of endpoints

The 30-day death rate was our main result, and the 90-day and 1‑year mortality rates were our secondary results. All participants were tracked for at least 1 year beginning on the day they were admitted in the study. The Social Security Death Index entries were used to determine the date of death.

### Statistical analysis

Three phases made up the statistical analysis method. The individuals were first separated into groups based on their baseline BMI. The Kruskal–Wallis *H* test or analysis of variance were used to compare means and standard deviations for continuous data. Categorical data were shown as frequency or percentage, and the analysis of chi-square test and Fisher’s exact test results was used to compare them. Second, the relationship between BMI and mortality was estimated using a Cox proportional hazards regression model with a 95% confidence interval (CI). As a reference group, those whose weight was normal (BMI: 18.5–25 kg/m^2^) were taken into account. Models 1 and 2 did not adjust for age, sex, or ethnicity; model 3 adjusted for age, sex, and ethnicity as well as for SBP, heart rate, SPO_2_, CHD, AF, DM, AKI, CHF, APACHE III, and SOFA, among other factors. Based on changes in the impact estimate of more than 10%, the confounders were chosen [24, 25]. Thirdly, a non-linear association between BMI and 30-day mortality was discovered using smooth curve fitting (penalized spline approach). The threshold impact of BMI on 30-day mortality was investigated using a two-piecewise linear regression model. Recursive analysis was used to pinpoint the BMI inflection point at which the association between BMI and 30-day mortality started to reverse.

The SPSS software (v22.0; IBM, Armonk, NY, USA) was used for statistical analysis. A two-sided *p*-value of < 0.05 was regarded as statistically significant.

## Results

### Participant characteristics

The baseline characteristics of the study participants are shown in Table 1. There were 185 women and 233 men (age: 70.7 ± 44.1 years; BMI: 28.7 ± 8.1 kg/m^2^). Compared with patients with normal weight, obese group comprised younger patients (60.1 ± 13.7, *p* = 0.003) and a higher percentage of female patients (51.3% vs. 49.0%, *p* = 0.001). There were significant differences in the distribution of DM, DBP, SBP, HR, GCS, and mortality across the groups with different BMI values.

**Table 1:** Characteristics of the study patients according to BMI

Variables	Underweight	Normal weight	Overweight	Obese	*p*
**Baseline characteristics**					
*N*	22	143	103	150	–
*Age, years*	72.4 ± 53.8	77.5 ± 53.4	76.3 ± 53.1	60.1 ± 13.7	0.003
*Sex, n (%)*	–	–	–	–	0.001
Female	4 (18.2)	70 (49.0)	34 (33.0)	77 (51.3)	–
Male	18 (81.8)	73 (51.0)	69 (67.0)	73 (48.7)	–
*Ethnicity, n (%)*	–	–	–	–	0.256
White	14 (63.6)	97 (67.8)	82 (79.6)	97 (64.7)	–
Black	2 (9.1)	13 (9.1)	6 (5.8)	18 (12.0)	–
Other	6 (27.3)	33 (23.1)	15 (14.6)	35 (23.3)	–
**Vital signs within 24** **h of ICU admission**					
*Heart rate, bpm*	77.2 ± 14.2	77.8 ± 14.8	81.0 ± 14.3	82.1 ± 15.3	< 0.001
*SBP, mm* *Hg*	125.3 ± 16.3	125.6 ± 17.5	124.0 ± 19.0	125.0 ± 18.7	0.037
*DBP, mm* *Hg*	62.6 ± 10.6	62.0 ± 10.9	60.8 ± 11.6	61.6 ± 11.1	0.005
*SPO*_*2*_*, %*	92.5 ± 2.1	92.5 ± 2.2	91.4 ± 2.8	92.4 ± 2.7	0.238
**Severity of organ dysfunction**					
*SOFA*	5.7 ± 3.2	5.5 ± 3.3	5.5 ± 3.4	5.5 ± 3.3	0.678
*GCS*	13.4 ± 2.5	13.9 ± 2.5	13.9 ± 2.4	13.1 ± 3.2	0.033
*APACHE III*	51.5 ± 13.6	52.0 ± 17.3	49.5 ± 21.2	55.7 ± 23.2	0.106
**Comorbidities**					
*CHD, n (%)*	–	–	–	–	0.163
No	19 (86.4)	105 (73.4)	77 (74.8)	124 (82.7)	–
Yes	3 (13.6)	38 (26.6)	26 (25.2)	26 (17.3)	–
*AF, n (%)*	–	–	–	–	0.059
No	17 (77.3)	96 (67.1)	73 (70.9)	121 (80.7)	–
Yes	5 (22.7)	47 (32.9)	30 (29.1)	29 (19.3)	–
*CHF, n (%)*	–	–	–	–	0.384
No	17 (77.3)	108 (75.5)	77 (74.8)	124 (82.7)	–
Yes	5 (22.7)	35 (24.5)	26 (25.2)	26 (17.3)	–
*Hypertension*	–	–	–	–	0.303
No	21 (95.5)	125 (87.4)	92 (89.3)	125 (83.3)	–
Yes	1 (4.5)	18 (12.6)	11 (10.7)	25 (16.7)	–
*DM*	–	–	–	–	0.006
No	16 (72.7)	128 (89.5)	84 (81.6)	111 (74.0)	–
Yes	6 (27.3)	15 (10.5)	19 (18.4)	39 (26.0)	–
*AKI*	–	–	–	–	0.176
No	7 (31.8)	25 (17.5)	17 (16.5)	20 (13.3)	–
Yes	15 (68.2)	118 (82.5)	86 (83.5)	130 (86.7)	–
**Mortality, *n* (%)**					
*30-day*	–	–	–	–	0.019
No	13 (59.1)	108 (75.5)	87 (84.5)	125 (83.3)	–
Yes	9 (40.9)	35 (24.5)	16 (15.5)	25 (16.7)	–
*90-day*	–	–	–	–	0.006
No	9 (40.9)	93 (65.0)	79 (76.7)	107 (71.3)	–
Yes	13 (59.1)	50 (35.0)	24 (23.3)	43 (28.7)	–
*1‑year*	–	–	–	–	0.017
No	7 (31.8)	72 (50.3)	65 (63.1)	90 (60.0)	–
Yes	15 (68.2)	71 (49.7)	38 (36.9)	60 (40.0)	–

Data are presented as mean ± SD and *n* (%)

Statistically significant differences between groups at* p* < 0.05

*SBP* systolic blood pressure, *DBP* diastolic blood pressure, *SPO*_*2*_ percutaneous oxygen saturation, *CHD* coronary heart disease, *CHF* chronic heart failure, *AF* atrial fibrillation, *DM* diabetes mellitus, *SOFA* Sequential Organ Failure Assessment, *GCS* Glasgow Coma Scale

### Obesity and prognosis in ARDS patients

Effect sizes of the association between BMI and ARDS outcome are shown in Table 2. During the follow-up period, 85 fatalities for 30-day mortality occurred. Model 1 is an unadjusted model, and when compared to people of normal weight, the 30-day mortality rates for underweight, overweight, and obese people were 1.57 (0.76, 3.27), 0.64 (0.39, 1.08), and 4.83 (2.25, 10.35), respectively. Similar trends were seen for 30-day mortality in model 2 after accounting for age, sex, and ethnicity, and the risk was more pronounced with greater BMI. Model 3 adjusted for age, sex, and ethnicity as well as SBP, heart rate, SPO2, CHD, AF, DM, AKI, CHF, APACHE III, and SOFA. The hazard ratio (HR; 95% CI) of 30-day mortality for the underweight, overweight, and obese patients was 1.82 (0.85, 3.90), 0.59 (0.29, 1.20), and 3.85 (1.73, 8.57), respectively.

**Table 2:** Hazard ratios (95% CI) for mortality across groups of BMI

RDW	Model 1	Model 2	Model 3
	HR (95% CI); *p*	HR (95% CI); *p*	HR (95% CI); *p*
**30-Day all-cause mortality**			
RDW (per 0.1 change)	0.95 (0.92, 0.98); 0.0014	0.95 (0.92, 0.98); 0.0043	0.95 (0.92, 0.99); 0.0080
*BMI classifications*			
Underweight	1.57 (0.76, 3.27); 0.2252	1.50 (0.71, 3.19); 0.2875	1.82 (0.85, 3.90); 0.1221
Normal weight	Ref.	Ref.	Ref.
Overweight	0.64 (0.39, 1.08); 0.0927	0.58 (0.32, 1.06); 0.0743	0.59 (0.29, 1.20); 0.1455
Obese	4.83 (2.25, 10.35); < 0.001	4.29 (1.93, 9.53); 0.0004	3.85 (1.73, 8.57); 0.0019
**90-Day all-cause mortality**			
RDW (per 0.1 change)	0.96 (0.94, 0.99); 0.0057	0.96 (0.94, 0.99); 0.0098	0.97 (0.94, 1.00); 0.0250
*BMI classifications*			
Underweight	1.72 (0.94, 3.17); 0.0808	1.52 (0.80, 2.86); 0.1983	1.94 (0.93, 2.89); 0.2095
Normal weight	Ref	Ref	Ref
Overweight	0.62 (0.38, 1.01); 0.0538	0.60 (0.37, 1.01); 0.0502	1.18 (0.78, 1.80); 0.4347
Obese	3.62 (1.76, 7.45); 0.0005	3.90 (1.84, 8.31); 0.0004	3.01 (1.42, 6.39); 0.0041
**One-year all-cause mortality**			
RDW (per 0.1 change)	0.98 (0.96, 0.99); 0.0097	0.98 (0.95, 0.99); 0.0108	0.96 (0.94, 0.99); 0.0064
*BMI classifications*			
Underweight	1.60 (0.92, 2.80); 0.0983	1.63 (0.92, 2.92); 0.0957	1.96 (1.09, 3.52); 0.0239
Normal weight	Ref	Ref	Ref
Overweight	0.67 (0.45, 1.00); 0.0480	0.71 (0.47, 1.06); 0.0942	0.79 (0.56, 1.13); 0.2044
Obese	3.01 (1.49, 6.09); 0.0021	3.27 (1.58, 6.78); 0.0014	2.84 (1.38, 5.82); 0.0044

Model 1: no covariates were adjusted

Model 2: covariates were adjusted for age, sex, and race

Model 3: covariates were adjusted for age, sex, ethnicity, SBP, heart rate, SPO_2,_ CHD, AF, DM, AKI, CHF, APACHE III, and SOFA

Statistically significant differences between groups at* p* < 0.05

*HR* hazard ratio, *CI* confidence interval, *Ref.* reference

A similar pattern was also seen for 90-day and 1‑year mortality, and the risk was more pronounced in patients with obesity.

**Fig. 1:**
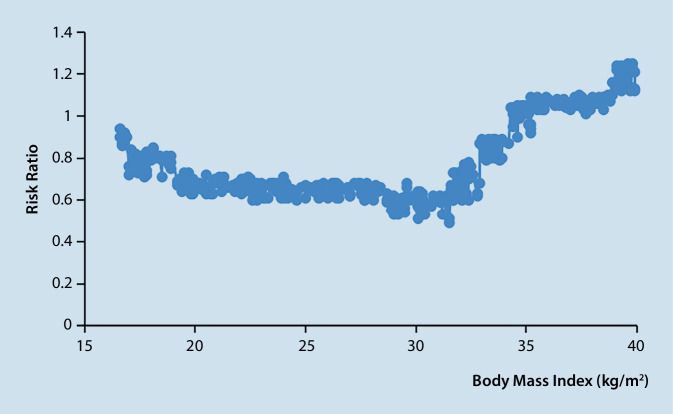
The relationship between 30-day mortality and body mass index in patients with respiratory distress syndrome

In order to show the nonlinear relationship between BMI and 30-day mortality, we fitted a smooth curve. Nonlinear relationships were seen after adjusting for other confounding factors. A two-piecewise linear regression model was developed due to the limitations of classification analysis, and the inflection point for BMI was 31.5. Increased BMI was linked to a lower risk of death in ARDS patients on the left of the inflection point (BMI 31.5), with an HR of 0.76 (95% CI: 0.60, 0.83); *p* < 0.0001. Increased BMI was linked to an increased mortality rate in ARDS patients on the right of the inflection point (BMI 31.5; HR of 1.08 (95% CI: 1.03, 1.11); *p* = 0.3385; Table 3; Fig. 1).

**Table 3:** Results of standard linear regression model and two-piecewise linear regression model

	HR (95% CI)	*p*
**Fitting model by standard linear regression**	0.95 (0.92, 0.99)	0.0080
**Fitting model by two-piecewise linear regression**		
*Inflection point of RDW*	31.5	–
≤ 31.5	0.76 (0.60, 0.83)	< 0.0001
> 31.5	1.08 (1.03, 1.11)	0.0179
*p for log likelihood ratio test*	< 0.001	–

Adjusted: age, sex, ethnicity, SBP, heart rate, SPO_2,_ CHD, AF, DM, AKI, CHF, APACHE III, and SOFA

Statistically significant differences between groups at* p* < 0.05

*HR* hazard ratio, *CI* confidence interval

## Discussion

After controlling for confounding variables, we discovered that, compared to normal weight, obesity was independently related to risk of both short- and long-term death. Additionally, there was a U-shaped association between BMI and ARDS mortality. A BMI over 31.5 kg/m^2^ was associated with a slightly increased risk of death. Between ARDS patients who are underweight and those who are of normal weight, the risks of death were not significantly different.

The findings of epidemiological studies that showed how obesity affected the outcome of patients with ARDS have been debatable so far [26–28]. Increasing BMI was initially associated with a decrease in mortality in patients with end-stage renal illness, according to Fleischmann et al. in 1999 [29]. Additionally, this link was seen in CHF, CHD, COPD, and critical illness [30–33]. The “obesity paradox” was the term given to the phenomenon. Jayanama et al. found that overweight (BMI 25.0–29.9 kg/m^2^) was a protective factor for mortality in people with moderate/severe debilitation people, and grade 1 obesity (30.0–34.9 kg/m^2^) was a protective factor for mortality in people with mild debilitation. By contrast, in the population without debilitation, class 2 or 3 obesity (≥ 35.0 kg/m2) may be associated with a higher risk of mortality [34].

According to a previous meta-analysis [26], individuals with obesity had greater ARDS morbidity than those who were of normal weight, and obesity was strongly linked to a lower ARDS fatality rate. Obese individuals are more likely to have comorbidities, including cardiovascular disease, respiratory conditions, and AKI; however, these studies did not account for these conditions. To support the conclusion, our research included detailed clinical, laboratory, and physiologic data. We made adjustments for a number of variables, including illness severity and cardiovascular risk, and we used curve fitting to fit the association between BMI and ARDS prognosis. The connection was seen to be U‑shaped. The two-stage study determined that 31.5 kg/m^2^ was the cutoff BMI.

The following might be the reason for our finding. First, ARDS development is significantly influenced by inflammation. Blood proinflammatory cytokines are higher in obese patients [35], which decreases antioxidant reserves, upregulates adhesion molecules on lung endothelium, and increases endothelium sensitivity to injury [36]. Second, an imbalance in adipokine secretion and reaction might result from obesity [35]. Adipokine imbalance, as shown by Shah et al. [37, 38], affected the expression of endothelial junctional adherens and adhesion proteins, predisposed the lung to acute damage, and disrupted pulmonary vascular homeostasis in obese mice. Finally, compared to people who are lean, patients who are obese undergo a number of modifications in pulmonary mechanics. The total lung capacity, functional residual capacity, and vital capacity are a few of the things that are altered in obese people. These changes also lead to atelectasis, increased airway resistance and closure, and ventilation/perfusion mismatch. These results firmly establish a connection between obesity and an increased risk of ARDS.

We further discuss the protective effects of a BMI of < 31.5 kg/m^2^ in patients with ARDS. Previous studies have found that a higher rate of body fat is associated with a higher degree of frailty after controlling for BMI [30]. Body fat rate has a mediating effect in the association of BMI with frailty, and the association of BMI with frailty in the vast majority of patients can be explained by higher body fat. But an increase in BMI does not exclusively mean an increase in body fat content. Body weight consists of fat mass, muscle mass, bone mineral mass, and body moisture. The increase in BMI may also be caused by an increase in these body components [39]. Studies have shown better quality of life in populations with high skeletal muscle mass and strength, with significant reductions in infection rates, hospital stay, immobilization, and mortality [40]. It is therefore reasonable to speculate that the protective effect in patients with BMI < 31.5 kg/m^2^ may be due to increased fat free mass.

There were several advantages in the present study. In order to ensure that the population included in the research was fairly representative and the findings were somewhat trustworthy, we first employed a database with a large multicenter sample for rigorous screening. Second, we employed a number of models to successively account for the impact of several known confounders on ARDS, including age, sex, comorbidities, heart rate, blood pressure, and other parameters. These models revealed a consistent correlation between BMI and sepsis survival. Finally, a two-segment linear regression model was used to examine the threshold effect of BMI on 30-day mortality in order to more thoroughly investigate the relationship between BMI and ADRS mortality than was done in earlier studies. The association between BMI and 30-day mortality was analyzed by smooth curve fitting (penalized spline method).

Nevertheless, there were limitations to be acknowledged. First, because this was a retrospective cohort research, it was unable to establish a link between BMI and death. Second, as with any cohort analysis, we attempted to control for potential risk factors including CVD, comorbidities, and others; nevertheless, residual confounders, such as proinflammatory variables, marital status, and other known or unknown confounders, cannot be fully ruled out. Third, only the first 24 h after admission were considered for BMI; the prognosis was not examined in connection to later BMI changes. Using only baseline evaluation raised the possibility of categorization bias.

## Conclusion

In conclusion, BMI was a reliable predictor of both short- and long-term mortality in patients with ARDS. There is a U-shaped relationship between BMI and ARDS mortality. A BMI over 30.5 kg/m^2^ was associated with a slightly increased risk of death.

## Declarations

All procedures performed in studies involving human participants or on human tissue were in accordance with the ethical standards of the institutional and/or national research committee (ethics committee of Zhuji People’s Hospital Affiliated to Shaoxing University of Arts and Sciences) and with the 1975 Helsinki declaration and its later amendments or comparable ethical standards. Informed consent was obtained from all individual participants included in the study.
